# Death as the Cessation of an Organism and the Moral Status Alternative

**DOI:** 10.1093/jmp/jhad018

**Published:** 2023-05-03

**Authors:** Piotr Grzegorz Nowak

**Affiliations:** Jagiellonian University, Krakow, Poland

**Keywords:** definition of death, moral status, brain death, organism, entropy, organ transplantation

## Abstract

The mainstream concept of death—the biological one—identifies death with the cessation of an organism. In this article, I challenge the mainstream position, showing that there is no single well-established concept of an organism and no universal concept of death in biological terms. Moreover, some of the biological views on death, if applied in the context of bedside decisions, might imply unacceptable consequences. I argue the moral concept of death—one similar to that of Robert Veatch—overcomes such difficulties. The moral view identifies death with the irreversible cessation of a patient’s moral status, that is, a state when she can no longer be harmed or wronged. The death of a patient takes place when she is no longer capable of regaining her consciousness. In this regard, the proposal elaborated herein resembles that of Veatch yet differs from Veatch’s original project since it is universal. In essence, it is applicable in the case of other living beings such as animals and plants, provided that they have some moral status.

## I. INTRODUCTION

Although more than 50 years have passed since the Ad Hoc Committee (1968) report, the publication of which launched the history of the modern neurological criteria of death, the criticism concerning the determination of brain death has become more intense over the last 20 years. The publications of Alan Shewmon, who has pointed out that brain-dead patients might constitute integrated organic wholes (2001), and that they are sometimes capable of persisting in such states for periods of over a week or even many years (1998), are to a large extent the primary source of these intense controversies. Shewmon’s findings fundamentally undermined the belief that brain death is the death of a human organism, a direct challenge to the opinion held by most academics working in the field.^1^ For them, the notion of “death” is a biological one, and all living organisms die in the same sense (see, e.g., Bernat, Culver, and Gert, 1981; Singer, 1994; President’s Council, 2008, 51–52). According to this view, brain death was perceived to be an adequate criterion of death, since it was believed that humans in such a state could not constitute an integrated whole, but merely a set of artificially sustained organs which will soon gradually deteriorate (Korein, 1978, 26–27; Bernat, Culver, and Gert, 1981, 391). In light of Shewmon’s analysis, this statement has been proven to be false.

Taking into account the dead donor rule, which forbids the retrieval of organs before the natural death of a donor (Robertson, 1999; Veatch, 2003; President’s Council, 2008, 100; Bernat, 2013; Nair-Collins, Green, and Sutin, 2015), and the fact that the brain-dead are the main source of organs all over the world (Global Observatory on Donation and Transplantation, 2018), an impasse has occurred. Three leading solutions were proposed to resolve this problem. First, it was put forward that brain death does not constitute the real death of a human being and, therefore, all types of conduct that are typically used with the truly deceased, such as organ retrieval, should be forbidden if they target those who are merely brain-dead (e.g., President’s Council on Bioethics, 2008, 52–58; Symons and Chua, 2018). The second line of argumentation ran that, even if brain death is not “real” death, organs should still be retrieved, provided that the patients gave their informed consent for such a lethal procedure (e.g., Miller and Truog, 2012; Nair-Collins, 2018b). Third, a distinction was proposed between the biological and moral sense of “death,” pointing out that in some contexts such as organ donation, the second sense is what matters (e.g., Veatch, 1975; Shewmon, 2010; Lizza, 2011; Veatch, 2015; Veatch, and Ross, 2016, 153).^2^

The first of the proposed solutions almost eliminates the possibility of organ transplantation, a therapy that saves many lives each year, and at least in the case of kidney transplantation is cheaper and more effective than all of the available alternatives (Jensen, Sørensen, and Petersen, 2014). Moreover, it requires us to treat the brain-dead as living people in all other respects. The second possibility demands social consent for causing the death of patients at their request, and might be accompanied by a decrease in the number of transplants (Nair-Collins, Green, and Sutin, 2015, 301). The third option seems the most promising, both theoretically and practically. Later on in this paper, I will elaborate on the theoretical merits of this solution. For now, it should be mentioned that it is welcomed for practical reasons: it does not require any changes to be made to our conduct (e.g., when it comes to organ transplantation), by simply providing a new concept of death that justifies why brain death is “real” death.

At present, there are almost no institutional bodies (such as the President’s Commission or President’s Council) that would refer to the concept of death as an irreversible loss of moral status as the main justification for equating brain death with death in their legislative documents. A frequently stated claim is that any legally acceptable concept of death must be based on the philosophy (theory) of biology, since biology and the other natural sciences provide “firm” knowledge which keeps the debate at a safe distance from philosophical discussions on unresolvable problems (e.g., Grisez, and Boyle, 1979, 75–8; Bernat, Culver, and Gert, 1981; President’s Commission, 1981, 39–40; Lamb, 1985; Bernat, 2002, 2006; President’s Council on Bioethics, 2008, 51; Marquis, 2010, 28, 2018; Miller, and Truog, 2012, 89; Condic, 2016).^3^ However, the strategy of being so attached to that kind of biological paradigm is based on dubious modifications to the concept of death itself, which do not stand up to criticism (Nair-Collins, 2018a, 35–39). In this article, I contribute to the discussion by providing an argument that indirectly favors the third option in the debate: I claim that the dominant biological concept of death is based on controversial assumptions and thus has no advantage over the moral account.

## II. THE BIOLOGICAL PARADIGM

The history of the justification of the neurological criteria of death which utilizes the biological concept of an organism began in 1981 in two influential publications: the article “On the Definition and Criterion of Death” by Bernat, Culver, and Gert and in the report “Defining Death” issued by the President’s Commission. Shortly after the publication of these texts, the neurological criteria were legally established in all states of the United States and most other countries. The justification for the adoption of these criteria was often similar to that which appeared in the aforementioned publications. Bernat, Culver, and Gert write about death in the following manner:

Death is considered a biological occurrence not unique to humans, and other higher animals would be considered dead according to the same definition. As a biological phenomenon, death should apply equally to related species. When we talk of the death of a man we mean the same thing as we do when we talk of the death of a dog or a cat. (1981, 389)

While, in the President’s Commission report, the following lines are included:

(…) death is that moment at which the body’s physiological system ceases to constitute an integrated whole. Even if life continues in individual cells or organs, life of the organism as a whole requires complex integration, and without the latter, a person cannot properly be regarded as alive. (1981, 33)

After the publication of these texts, scores of bioethicists frequently ventured the thesis that the word “death” has a biological meaning, and that different living organisms die in the same sense. Even proponents of the moral approach admit that “death,” understood as an irreversible loss of moral status, might only be a technical concept, whilst the biological sense of “death” would be the primary notion (McMahan, 2002, 425). Abandoning the field in this way to the proponents of the biological paradigm overlooks the fact that biology, as well as considerations on the margins of life, lacks a unified definition of an organism (see, e.g., Pepper and Herron, 2008; Nicholson, 2014; Brown, 2019; Kingma, 2019, 623–631). This is a crucial problem for biological concepts of death, which are based on either the President’s Commission report or the famous article of Bernat et al. The linchpin of the argument advanced by the proponents of such paradigms, according to which the word “death” in the biological context has a clearly determined meaning, as opposed to “death” in the moral sense, collapses. If we do not have clarity concerning the meaning of the word “organism,” if the meaning of the term “living body” is muddled, then we cannot precisely define death in a biological sense (Magnus, 2018).

### An Integrated Whole

In the context of the debate over brain death, the only consensus about the meaning of the word “organism” achieved to date is very humble and amounts to the statement that an organism constitutes an integrated whole or an integrated unity, yet this qualification is a nebulous one at best.^4^ One of the few proposals to operationalize it was provided by Shewmon, who formulated the following criteria to distinguish between organisms and things which are dead or inanimate^5^;

CRITERION 1. “Integrative unity” is possessed by a putative organism (i.e., it really is an organism*) if the latter possesses at least one emergent, holistic-level property*. A property of a composite is defined as “emergent” if it derives from the mutual interaction of the parts... and as “holistic” if it is not predicable of any part or subset of parts but only of the entire composite. (2001, 460)

The fulfillment of this criterion is necessary for one to be a living human organism, yet it is not sufficient because, as its author notices, it might be satisfied by entities that are certainly not organisms, such as societies (Shewmon, 2001, 461). According to Shewmon, something more is needed for a given object to be a living human organism:

CRITERION 2. Anybody requiring *less* technological assistance to maintain its vital functions than some other similar body that is nevertheless a living whole must possess at least as much robustness of integrative unity and hence also be a living whole. (2001, 462)

Shewmon develops the first and second criterion as necessary and sufficient conditions for a given object to be classified as a living organism. However, the second criterion allows one to distinguish living organisms only if one already knows that some individuals under artificial support are living organisms at the outset. As a result, it is disappointing. It requires us to either refer to some other criteria permitting patients to be determined as biologically living at the outset, or to agree that all living patients (irrespective of their health condition) are identified as such intuitively without having in common any single characteristic (Chiong, 2005; Żuradzki and Nowak, 2019; see also Shewmon, and Shewmon, 2004, 90–91).

### Alternative Criteria

How might the first criterion provided by Shewmon be supported to separate the subcategory of living organisms from the set of other individuals which constitute integrative unities? The first idea came from Shewmon himself, who, while referring to Varela (1979), writes that “... living organisms topologically demarcate «self» from «non-self» by a continuous, closed membrane...” (2001, 461). This proposal is far from persuasive since artifacts made by humans, such as computers, are sometimes in possession of “a continuous, closed membrane” and have at least one holistic and emergent property. The capacity to compute constitutes precisely such a property. It cannot be predicated on any part of the device, but only about the computer as a whole (it is holistic), and it is an effect of the integrated coordination of its parts, that is, processor, RAM, and input/output devices (it is emergent). Consequently, computers satisfy the first criterion proposed by Shewmon, and are equipped with “membranes” separating them from the external world. Therefore, if Shewmon’s first criterion, together with the membrane criterion, are proposed as necessary and sufficient criteria for being an organism, it seems that computers are organisms even though (quite naturally) no biologist would recognize them as such.

One could try to defend this way of differentiating organisms from inanimate and dead material by reference to Varela’s other idea, namely to the notion of autopoiesis:

an autopoietic system is organized (defined as a unity) as a network of processes of production (transformation and destruction) of components that produces the components that: (1) through their interactions and transformations continuously regenerate and realize the network of processes (relations) that produced them; and (2) constitute it (the machine) as a concrete unity in the space in which they exist by specifying the topological domain of its realization as such a network. (1979, 13)

One might claim that for a given entity to be an organism, it not only needs to possess at least one holistic and emergent property, and to be equipped with a membrane, but it also needs to be an autopoietic system.^6^

There are, however, serious problems with utilizing autopoiesis as an eligibility criterion for being an organism. Pier Luigi Luisi (one of Varela’s co-workers) noticed that the notion of autopoiesis and the concept of life that is associated with it is quite nebulous and lies rather on the margins of science (2003; c.f. Bourgine and Stewart, 2004). The fuzziness of the notion of autopoiesis endangers the very goal of Shewmon’s analysis, which was among others to provide a “careful operational definition of terms” (2001, 459) that are present in the definition of death debate. Since autopoiesis is not straightforward, it is unclear which entities are to be classified as autopoietic, and therefore living. At the very beginning, this concept was invented to describe mechanisms of only cellular life. As Luisi noticed, it took a long time for Varela to accept that autopoiesis might be generalized to higher organisms (2003, 52). Yet, some social scholars uphold that social systems such as families and political parties also might be perceived as autopoietic (see, e.g., Teubner, 1993; Luhmann, 1995; Mingers, 1995, 1997). Are such social systems themselves living organisms? Such a conclusion seems to be dubious.

Moreover, even if the criterion of autopoiesis removes computers as members of the family of the living since they do not regenerate their hardware, it might still allow for some human-made artifacts to be recognized as organisms. The first example of such an object is a computer program designed by Varela et al. According to Varela et al., the program, if run, is capable of fulfilling the minimal criteria for autopoiesis, and of possessing at least one holistic and emergent property (1974; McMullin and Varela, 1997).^7^ The second example is what is currently called “wet artificial life” (Badeau, 2003; Luisi, 2003; Sulllins, 2005; Fellermann, 2011), which is a kind of self-reproducing system of molecules obtained by chemists in their laboratories.

### The Entropic Concept of Death

Since the notion of an “integrated whole” that is used to define an organism seems to be too nebulous to constitute a scientific basis for such a definition, one could try to find a way to provide a more science-informed method of distinguishing living organisms from inanimate and dead material. Such an idea might be sought in the notion of entropy, which has been utilized to define life since the publication of the famous book “What is Life” by Erwin Schrödinger. Scientists often explain what entropy is, saying that it is a measurement of disorder (Lambert, 2002, 187–188). However, such an explanation is only a crutch. More precisely, entropy is described by Franklin Lambert in the following way:

... “entropy change measures energy’s dispersion at a stated temperature.” This concept of energy dispersal is not limited to thermal energy transfer between system and surroundings. It includes redistribution of the same amount of energy in a system—for example, when a gas [or liquid] is allowed to expand into a vacuum container, resulting in a larger volume. (2002, 187–188)

According to H theorem, every isolated system of molecules (a system with no entrance and no exit) will unavoidably tend toward a state of maximum entropy. When it reaches this state, it will never deviate from it (cf. Brown, Myrvold, and Uffink, 2009). If H theorem is true, and if the whole world is an isolated system as some scientists suppose, then living organisms are a very peculiar fragment of it:

... [they] are localized pockets of anti-entropy, achieved by mutually interdependent functional structures jointly maintaining internal equilibrium, or homeostasis of the extracellular fluid, a necessary condition for all organismic function, while resisting chemical and thermal equilibrium with the external environment. (Nair-Collins, 2018a, 32)

Along the lines of this entropic definition of life, several versions of the entropic view on death might be distinguished. Here I will focus on one which appears commonly in the context of the brain death debate (Korein, 1978; Shewmon, 2001; Miller and Truog, 2012; Bernat, 2019; Huang and Bernat, 2019). According to this concept, the characteristic which differentiates living organisms from other parts of the world is the fact that organisms are capable of opposing entropy within themselves, and they die when they irreversibly lose this capacity. An example provided by Michael Nair-Collins might help to clarify what is usually meant by the capability to oppose entropy increase:

... A trip and fall in an otherwise healthy thirty-year-old might create a bruise or scraped skin, but would not pose too much of a threat. But a fall in a very old person, especially someone who is frail, is a grave threat to their health and even life. It often signals the beginning of their functional decline, because their physiologic reserve is much lower. Taking the idea of homeostenosis to its natural conclusion, when the organism no longer has the ability to restore homeostasis, the organism has died. (2018a, 32)

How to understand what happens here in terms of entropy? Roughly this way: when a 30-year-old falls, homeostasis is disturbed for a while, meaning that entropy increases in their organism (energy disperses). But the bodies of healthy 30-year-olds usually have the requisite physiological reserves, with some access to external resources, that make them able to reduce the level of entropy, and restore homeostasis. Such unfortunate accidents generally end in just a bruise or scraped skin. The upshot is that the ability to reduce the level of entropy or, in other words, restore homeostasis, is to be understood as being synonymous with life.^8^ But what is death according to such a view? Miller and Truog have stated this perhaps most explicitly: “... death occurs at the moment when the entropy-increasing forces have irreversibly exceeded those that are resisting this process.” (2012, 70). I suppose that Miller and Truog understand death just as Nair-Collins does—as a state when an organism has not enough physiological reserves to restore homeostasis.^9^

Even though the entropic concept of death seems to be more theoretically sophisticated than the concept of death as a loss of somatic integration, it is still disappointing, at least if we would like to adopt such a concept for purposes other than scientific ones, such as healthcare. There is no method for actually measuring entropy in an organism, nor even any hypothetical understanding of what a numerical measurement of entropy in an organism would even mean. We can only speculate philosophically on measures of entropy in organisms, and it seems that everyone can do so as she pleases. Taking into account the vagueness of this concept, academics on opposing sides of the debate can easily refer to the entropic view of death arguing for their position: some of them claim that it justifies brain death as a criterion of death (Korein, 1978; Bernat, 2019; Huang and Bernat, 2019) and others claim that it explains why brain death is not an appropriate criterion of death (Shewmon, 2001; Miller and Truog, 2012).

Another problem with the entropic concept of death is that it might have absurd consequences, at least if we make some assumptions about the measurement of entropy in organisms. Let me recall that according to this view, an organism dies when its physiological reserves are insufficient to restore homeostasis. To return to Nair-Collins’ case mentioned above, suppose that the one who fall suffers from hemophilia, and the accident causes her to transect one of her main arteries. If death is seen as a moment when one has no reserves to restore homeostasis, it might be the case that such a person “dies” before she loses consciousness. It might be impossible for the hemophiliac to restore homeostasis with her physiological reserves if there are no people around to help and, in such circumstances, entropy irreversibly increases.^10^ Nevertheless, even if physiological reserves are absent, we would like to say that people in this kind of situation are in some sense alive (they are conscious!). While considering such cases, we understand that, although the modern entropic version of the concept of death might seem to cohere well with some scientific theory, it is not the concept of death that we are looking for in the context of human conduct and social relations.

### The Fuzzy Concept of an Organism

One might wonder why bioethicists upholding the belief that death is the cessation of an organism generally do not utilize the works on the concept of an organism that are present in the philosophy of biology.^11^ One reason might be historical: philosophers of biology in the 1980s, when the “biological” status quo in the definition of death debate emerged, were not interested in the concept of an organism, focusing instead on populations and genes (Nicholson, 2014). Some of them even questioned whether organisms exist (Ruse, 1989). When, at the beginning of the 21st century, they finally focused their attention on the theory of organisms, they realized that no universal concept of an organism is present in natural sciences. Rather there is a plurality of such concepts, depending on the character of the given scientific research (Pepper and Herron, 2008; Nicholson, 2014; Stencel and Proszewska, 2018, 613–617; Kingma, 2019, 623–631). Ellen Clark perhaps makes this point most forcefully:

Biologists rely heavily on the concept of the organism, but they import different concepts into their models and discussions without reaching a consensus about which concept should be used, and usually without even being aware that they are talking about different things. (2010, 323)

Even though from the beginning of the new century the philosophy of biology has focused on the concept of an organism (Nicholson, 2014), bioethicists, upholding the “biological” definition of death, usually do not utilize the findings of philosophers of biology (Nowak and Stencel, 2022). We might observe a lack of intersection points of bioethical and philosophy of biology debate because bioethicists working in the field of brain death debate believe that there is only one well-established concept of an organism, assuming that death might happen only once (cf. Shewmon and Shewmon, 2004). Yet if there is no such thing as a single universal concept of the organism, if there are multiple concepts, there might also be numerous biological deaths. But such a conclusion is not what bioethicists were looking for.

Thus far I have presented problems associated with the biological concept of death enshrined in the brain death debate. Now is the time for a concluding remark on this part of the discussion: efforts made to defend this version of the biological concept of death fail.^12^ Even though such a concept aspires to be scientific, it is detached from the notion of an organism that is present in theoretical biology. At the same time, the concept of an organism developed originally within the brain death debate is problematic. It is either based on the nebulous notion of integrative unity or it defines conditions for being an organism in terms of entropy, which is immeasurable in the case of the living. Recently, James Bernat realized that the “founding myth” of this version of the “biological” concept of death about the universal and univocal concept of an organism is naive. Bernat has confessed that “[w]e should not demand more rigor and specificity from theoretical biological formulations than their intrinsic limitations permit and keep these categorical limitations in mind as we note the imperfections of biophilosophical formulations of death.” (2019, 4). In the part of the article which follows, I will argue that in the usual circumstances of our ordinary life, the death of a particular human does not have much in common with the criteria for being an organism and that in our daily life circumstances we *should* think about death as a moral phenomenon.

## III. DEATH AS A MORAL RATHER THAN A PURELY BIOLOGICAL ISSUE

In the previous sections, I have pointed out several problems with the biological concept of death. The fact that we think that it would be bizarre to state that a conscious but inexorably dying patient is dead, reveals that something else than the biological concept is at stake. We come to the same conclusion when we find out that we are not satisfied with the idea that there are multiple equivalent biological deaths of a human. We feel that we need one concept of death for the purposes associated with healthcare. It is a mistake to understand life and death as fundamentally biological phenomena. Clearly, a theoretical analysis of “life” and “death” in a biological sense is an essential task for theorists of biology, but it is not this type of analysis that we are looking for when confronted with the decisions at the bedside.^13^

We should notice that healthcare is still a form of care. Even if the achievements of contemporary natural science inform it, it is not natural science itself. Nor is medical treatment a form of scientific conduct. The goal of healthcare is to care for patients and their well-being in a scientifically informed way, and perhaps to promote an understanding of the biological preconditions of patients’ well-being. Yet the natural sciences cannot help us with defining “life” and “death” in a sense which matters for practical decisions. Life and death are, in some sense, clearly ethical terms, not biological ones. That is the case when Thomas Nagel writes about life that “[it] is all we have and the loss of it is the greatest loss we can sustain” (1979, 1; cf. Baker, 2005, 16–17). Here he does not mean the loss of one’s status as an organism related to the particular research paradigm, but the loss of capacities “... like perception, desire, activity, and thought, [that] are so general as to be constitutive of human life” (Nagel, 1979, 2). According to Nagel, these capacities are good for the people who manifest them and are preconditions of whole human multidimensional well-being.

Robert Veatch, who has been involved in the debate on the definition of death almost from its very beginning, points out that when it comes to the determination of death in the context of bedside decisions, as well as for other public policy purposes, what is really at stake are “death behaviors.” Together with Lainie Ross, they observe that “[s]ocial and cultural changes take place when we label someone as dead.” (2016, 27). When death occurs, some behaviors become appropriate, such as the stopping of palliative treatment, the onset of scientific research on newly dead bodies, retrieval of organs for transplantation, mourning, reading a person’s will, disposing of a person’s “mortal remains,” assuming the role of widowhood or widowerhood, and perhaps much more (2016, 28). Theoretical biology, with its multiple concepts of an organism, cannot help us to establish when such behaviors are appropriate, and when they are not. This shows that in the context of bedside decisions, it is the moral rather than the biological sense of life versus death distinction that is at stake. Veatch and Ross observe that in this context, “death comes to mean... nothing more than the condition of some group of human beings for whom death behavior is appropriate” (2016, 31). Elsewhere Veatch, this time alone, defines “death” in the following way:

[It is] the name applied to the category of beings who no longer have full moral standing as members of the human community with all the rights of that community (including the right not to be killed). This is no longer a biological use of the term; rather, it is a moral and legal use, what I call the “social meaning of death.” One first identifies who it is who is no longer part of the community in the full sense, that is, those not protected by laws against homicide, those who no longer can claim health insurance, those for whom life insurance should pay off, those whose spouses appropriately assume widowhood, etc., and then calls that group dead by definition. (2015, 9)

Veatch believes that his definition of death leads to the identification of brain-dead patients as dead since, according to him, they lack “full moral standing” (2015, 10–11), and this is the reason why “death behaviors” become appropriate towards them.

While I sympathize with Veatch’s proposal, especially with his observation that what matters in the context of end-of-life care are not biological considerations on concepts of an organism but moral considerations on the ethical permissibility of some behaviors, I see two problems associated with his definition. First, just as Nair-Collins points out, “the mere assertion that normativity is analytically built-in to the concept of death, *without giving further reasons for why the brain-dead lack moral status,* is question-begging.” (2017, 542). I agree with this comment on Veatch’s idea, and I agree with Nair-Collins when he continues his criticism of Veatch’s position stating that “*[w]hether* brain-dead patients can be harmed or wronged, and *whether* they have significant moral standing, are precisely what is at issue” (2017, 542).^14^ Second, if we define death as a loss of full moral status as a member of the human community, death becomes something unique for humans, a consequence that is unwelcome at least for some scholars working in the field (Bernat, Clulver, and Gert, 1981; President’s Council, 2008, 97).^15^ In what follows, I will elaborate a concept similar to that of Veatch, and show how these two issues might be addressed without defining death in purely biological terms.

### *A* Moral Status and *the* Moral Status

Veatch has defined death by utilizing a concept of moral status. One who is unfamiliar with philosophical ethics might wonder what this concept concerns precisely. To clarify this issue, it is helpful to recall Mary Ann Warren’s characteristics of moral status:

[An] important feature of the concept of moral status is that the moral obligations that are implied by the ascription of moral status to an entity are obligations to that entity. To violate an obligation arising from A’s moral status is to wrong A, and not merely some third party. For instance, if A’s moral rights have been violated, then it is A that has in the first instance been wronged, and on whose behalf others may complain. (1997, 10)

An upshot is that entities that have moral status are objects of at least some *direct* obligations, as opposed to merely *indirect* obligations. What is the difference between objects of moral status towards which we have some direct commitments, and things that have no moral status at all but might be objects of indirect moral obligations, becomes apparent thanks to another passage from Warren:

Suppose that you go on vacation, leaving your house in the care of a friend, who then sells your kitchen appliances and absconds. A moral wrong has evidently been committed; but it is obviously a wrong against you and not against your stove and refrigerator, which do not have moral status. Had you, on the other hand, left your pet pig in the care of a friend, who then sold it to a meat packing plant, then it would have been less clear that a wrong had been committed only against you. And if you had left your baby with a friend, who then sold him or her to a black-market baby broker, almost no one would doubt that a wrong had been committed not only against you but also against the child. In each case, your friend wrongs you by selling something that ought not to have been sold under those circumstances; but only in the third case does that which is sold have a moral status that most people would agree precludes its being sold under any circumstances. (1997, 10)

Entities that have moral status have “something” that make them the objects of direct obligations. A child from Warren’s story had this “thing,” and thus had moral status while a kitchen had no such “thing,” and therefore it had no moral status at all. More formally, we might define moral status in the following way: a given entity has moral status if, and only if, there are some reasons to act for the sake of this entity or its interests (Jaworska and Tannenbaum, 2014, 2018). Furthermore, the abovementioned “thing,” which the child from Warren’s story had, and the kitchen did not have, might be called “a ground of moral status” (Jaworska and Tannenbaum, 2018).

Having defined moral status, and bearing in mind what has been said about the ethical version of the life versus death distinction in a previous section of the article, we are ready to solve the second problem associated with Veatch’s definition of death: why should we define death in a general moral sense as a loss of “full moral standing as members of the human community” excluding all non-human beings from the set of objects that might die? Given the analysis of the concept of moral status, it is apparent that death in a general moral sense might be defined in a different way than Veatch proposed: as an irreversible loss of A’s moral status (whatever A is precisely). Although Veatch is right in defining death in close connection with the sphere of moral conduct, he is wrong to focus solely on death behaviors towards humans. If there is something that constitutes a basis for a moral status of other beings than humans, there might be death also in the ethical sense of other entities than humans.

This brings us to the first problem of Veatch’s definition of death: what exactly constitutes the grounds of a moral status, and do the brain-dead have this “thing”? Although an in-depth analysis of the possible grounds of moral status would require another article focused solely on the moral concept of death,^16^ a good starting point is the eclectic view on moral status that has been developed by Warren. It is a good starting point since it takes into account almost all of the grounds of moral status that are present in the debate, and are usually seen as competing, and adopts them within a single pluralistic theory. Warren claimed that there are, roughly speaking, six “things” which might make an individual the object of some moral obligations: being alive (in the strictly biological sense, whatever it means), sentience, moral agency, positive impact on ecosystem, being a subject of social relations (especially being the kind of entity which might be cared for), and being an individual whose moral status is conferred by other individuals that have moral standing (1997, 3–177).^17^According to Warren, each of these criteria is a sine qua non for *some* sort of direct moral obligations toward a given entity to occur. Therefore, each criterion entails *some kind* of moral status for the object which fulfills it. Depending on the criterion in question, moral status might be of differing degrees of strength, ranging from the lowest moral status (perhaps in case of being just biologically alive), which binds us very weakly, to full moral status, which entitles moral rights (e.g., in the case of moral agency). Warren’s analysis of moral status should perhaps be supplemented with the recent developments within the discussion on the grounds of *full* moral status. Recently, Agnieszka Jaworska and Julie Tannenbaum have proposed a criterion of incompletely realized sophisticated cognitive capacities, such as an incomplete capacity to be self-conscious, as a sufficient basis for full moral status (2014, 2018). Jaworska and Tannenbaum’s criterion entails that even severely mentally disabled people have full moral status, including the right not to be killed.

If we consider the seven above-mentioned criteria of moral status, one can realize that the moral concept of death, as presented here, is not to be objected to on the basis that, according to it, death becomes something unique for humans.^18^ We can speak not only about the “moral” death of a human being but also about the “moral” death of a dog (since dogs are sentient beings), and about the death of a piece of seaweed (since they are living organisms). Let me again recall that death in a general moral sense is to be defined as an irreversible loss of A’s moral status whatever A exactly is.

So we can speak about the life and death of all entities, which might fulfill at least one of the seven criteria for moral status. But what exactly can we say about the status of a brain-dead individual from the perspective of such a theory? The apparent problem is that even corpses which have lain peacefully at rest in their graves for many years, not to mention the brain-dead, might have some of the properties which make them a subject of some moral status according to Warren’s list. They can possess moral status due to a social relation of care. For the sake of an example, we might assume that family members embalmed them and cared for the resulting mummies as if they were members of society, and the mummies “responded” adequately by avoiding putrefaction. Moreover, human corpses, as well as the dead bodies of other animals, undoubtedly have some positive impacts on the ecosystem, another criterion of moral status in Warren’s view. Their moral status might also be conferred by adult people who undoubtedly have moral standing, just like the moral status of Uluru, which is, according to Warren, conferred to a rock formation by aborigines who believe that Uluru is a sacred place (1997, 171). Does the fact that mummies fulfill some of Warren’s criteria entail that they are living (ethically) people?

Obviously not. According to the moral definition of death, as proposed herein, death is an irreversible loss of *A’s* moral status, whatever *A* exactly is. Such a definition not only requires an analysis of the possible grounds of moral status, a task that is already completed, but also calls for the consideration of what kind of moral status is one of *A’s*. In other words, death is not only the cessation of moral status, but a cessation of the moral status of a given entity *as such an entity*. We have to realize that some objects might lose *their* moral status completely, possessing at the same time some sort of moral standing which is, however, not that of *theirs*, but is a moral standing of something else (e.g., of their remains). Therefore the fundamental question associated with the ethical definition of death which needs to be answered in the context of healthcare is the question “who are *we?*” or in McMahan’s words, who is “one of *us?*”^19^ We need to know who are the entities which are patients with *their particular* moral status, the beings which might cease to be patients, and cease to possess a *patient’s* moral standing.

Jeff McMahan has persuasively argued that if we want to come to any reliable conclusion about who we are, we should start with the notion of egoistic concern, realizing that there is a vast difference between our concern for our well-being and our concern for the well-being of others. This intuitive difference between the way we care for ourselves and the way we care for all other beings that are not to be identified with us should inform an analysis of our identity. To grasp the difference between egoistic and non-egoistic concern, it is helpful to recall Marya Schechtman’s words, who noticed that “we all know the difference between fearing for our own pain and fearing for the pain of someone else. The difference here consists not in *degree*—I may care more about the pain of my beloved than about my own—but in kind” (1996, 51–52). If we find a reliable account of what it is reasonable to care in an egoistic way, then we will also find a reliable account of “who *we* are” (McMahan, 2002, 51).

According to McMahan, if we test our intuitions with a series of thought experiments, we will conclude that *we* are embodied minds that are to be identified with the cerebral hemispheres that normally contain the content of our consciousness (2002, 3–94). We are not souls, nor organisms, nor minds detached from brains, since it is not rational to care in an egoistic way for such entities. Here I simply take it for granted that McMahan’s arguments about the model of egoistic concern are correct, since there is no space in this article to discuss them in much detail.^20^

An upshot of McMahan’s analysis for the discussion of human death is that, even if embalmed corpses have some sort of moral status due to the relation of care, and might, therefore, be in some ethical sense some kind of living entities, they plainly do not have the moral status of a human or of a “being like us” in McMahan’s terms, because the cerebral hemispheres in an embalmed bodies are irreversibly and totally destroyed. We do not have any strict egoistic reasons to care for the well-being of corpses, even if there is such a thing. Nor has anyone else any reason to care for our corpses in a way in which she cares for *us*. Of course, we might care for corpses in the broad sense of egoistic concern, just as we care about an inheritance and the fate of our heritage, but we do not care for such things as for ourselves but rather for something whose fate might have some indirect impact on our well-being.^21^

If McMahan’s arguments are correct, which I believe is the case, and human beings are embodied minds, then it is clearly visible that only two of Warren’s criteria are of particular importance—sentience and moral agency—since only these criteria concern mental characteristics. Taking into account McMahan’s arguments, Jaworska and Tannenbaum’s criterion of incompletely realized sophisticated mental capacities also remains in force, since to possess imperfectly realized sophisticated capacity such as self-consciousness one needs to have at least some sort of mental life such as sentience (2014, 2018; fns. 25 and 48). Accordingly, if a given entity fulfills neither of these three criteria, human moral status cannot be ascribed, even though perhaps in some circumstances we might award them moral status of a different kind, and therefore also a life (in the ethical sense of this word) of a different kind. This surprisingly shows that a set of living things in the ethical sense is not a subset of living organisms in a biological sense, but rather that living organisms are a subset of living objects in an ethical understanding. Talking about inanimate objects as subjects of life that might “die” at some point, seems at odds with the common parlance, although we sometimes do so, for example when we talk about the so-called “heat death of the universe” (cf. Brown, Myrvold, and Uffink, 2009). Yet, more importantly, the “ethical” meaning of life and death has been present in philosophical thought dating back to Plato, who believed that objects like a piece of iron or bronze might die (in the ethical sense) due to factors which are “bad” for them. In the case of a piece of iron or piece of bronze, Plato believed that rust might cause their death as a loss of their moral status (See the *Republic* 608d-609a, trans. Allan Bloom).

## IV. CONCLUSION

Are brain-dead patients alive or dead if we define human death as the irreversible loss of a human’s moral status? To answer this question, one needs to consider whether they—the patients—might regain their consciousness. I believe that the tests that are used for the determination of brain death (as opposed to clinical tests for the diagnosis of a permanent vegetative state, cf. Lizza, 2018a; fn. 26) allow us to determine with sufficient certainty that the cerebral hemispheres of a particular patient have been wholly and irreversibly destroyed, together with their capacity for sentience and moral agency.^22^ If this is the case, it might be concluded that brain-dead patients are really dead in the socially important sense of this word: they have lost their moral status. They are gone, although their remains might still be alive, both biologically (depending on the adopted concept of an organism) and ethically as objects of some moral status that is, however, not a moral status of patients but of some different kinds of beings.

Yet since this is a philosophical article and not a clinical one, the above conclusion is only conditional. In essence, it is based on the belief that a patient that is appropriately determined as brain-dead has cerebral hemispheres which have been irreversibly destroyed. If this condition is not met, then the whole conclusion does not follow. The recent case of Jahi McMath, a teenage girl who was diagnosed with brain death, and later had symptoms of a minimally conscious state (Shewmon, 2018a, 2018b; Truog, 2018), casts some doubts on this conclusion. Alan Shewmon hypothesizes that McMath had a global ischemic penumbra (GIP) that mimicked brain death. He states that currently “[t]here is no way to know what proportion of patients diagnosed BD might actually have been in GIP” (2018b, 169). If Shewmon’s hypothesis is right, and if he is correct in presuming that some subset of misdiagnosed patients have a “potential to improve to a level of MCS like Jahi, or possibly even higher,” then we should perhaps look for a different set of neurological tests for the determination of death. Taking into account the moral concept of death presented herein, such tests should be focused much more closely on the functioning of the cerebral hemispheres than current neurological tests for brain death.

## Footnotes

1. Note that James Bernat, who is one of the fathers of the biological concept of death, according to which human death is to be equated with the cessation of a human organism, perceives Shewman’s argument as not undermining his concept: “Alan Shewmon... who early in his career was a strong supporter of brain death, has shown inadequacies of the original rationale that the brain was the central integrator of the body... My long-standing defense of brain death as human death centers on the cessation of the human organism as a whole, a concept related to but distinct from bodily integration.” (2019, 2) Yet it is unclear if this is the case since, contrary to Bernat’s words, Shewmon states explicitly that his criticism of brain death as a death of a human being refers to the concept of death as the cessation of an organism as a whole. In his article, Shewmon (2001, 46–462) provides two criteria aimed at helping to distinguish living organisms from dead and inanimate objects, and this will be analyzed in this article.

2. These three options are obviously *the main* positions in the debate and are far from exclusive. Another possible stance emerges from recent writings on the role of values in science. Some philosophers argue that values (also non-epistemic ones) might influence scientists as reasons to adopt certain theories (e.g., Elliott, 2011). Along these lines, one could say that the mere biological theory of death is not value-neutral and cannot be. If science really has such value-laden nature, scientists should perhaps adopt the *biological* view on death, which is most beneficial overall. This might lead to the acceptance of brain death as a criterion for the death of a human organism. I would like to thank Tomasz Żuradzki for drawing my attention to this issue.

3. Here are a number of citations that show scholars working on a mainstream biological rationale for equating brain death with human death perceive their position as being based on a much firmer scientific basis than the moral concept of death: (1) the opinion about the concept of death as the loss of personal identity stated by the President’s Commission: “The argument from personal identity does not rely on any particular conception of personhood, but it does require assent to a single solution to the philosophical problem of identity. Again, this problem has persisted for centuries despite the best attempts by philosophers to solve it.” (1981, 39–40). I perceive these lines as implicitly suggesting that the notion of an organism is less philosophically controversial than the notion of personal identity, which is simply false as I will show in this article. One could argue that these lines refer to the notion of personal identity, not to the notion of moral status, but in the early days of the brain death debate, these two rationales were not kept separate, as evidenced by the President’s Commission references to Robert Veatch (1975) where he formulates his concept of death as the loss of moral status. (2) The opinion held by the President’s Council about the difficulties associated with John Lizza’s concept of death (which is strictly speaking a concept which equates death with the loss of both the status of a person and moral status) is as follows: “Thus, advocates of this... position effectively maintain that in certain cases there can be two deaths rather than one. In such cases, they argue, a body that has ceased to be a person (having ‘died’ the first death) can be treated as deceased—at least in certain ways.... Serious difficulties afflict the claim that something that can be called ‘death’ has occurred even as the body remains alive. One such difficulty is that there is no way to know that the ‘specifically human powers’ are irreversibly gone from a body that has suffered any injury shy of total brain failure.” These lines also implicitly suggest that it is easier to establish the concept of organism than the concept of “specifically human powers” (1981, 51). (3) Franklin Miller and Robert Truog venture the following opinion: “Consciousness is identified as the key to life and death by advocates of the higher brain standard because they see it is as essential to what it means to be a person. Although what it takes to be a person is far from clear, the ability to experience self and others—subjectivity and intersubjectivity—is generally thought to be an essential attributes. Accordingly, any consciousness-based standard for determining death necessarily parts company with a biological conception of death. Plants and lower animals are not persons, nor is the human fetus before the development of the neural capacity for consciousness. But these beings certainly are alive. Why should death be something essentially different on the human level than in the rest of the biological realm?” (2012, 89). This opinion also implicitly implies that the notion of an organism and the criteria for being an organism are much firmer than those for being a person. Incidentally, in this citation, we see another conflation of two separate concepts of death: death as a loss of status of a person with the notion of death as a loss of moral status.

4. For further criticism of the role of the notion of integrated unity and integrated whole see: Lizza (2018a, 2018b). For the exemplary formulation of the consensus in question in the context of the philosophy of biology, the end-of-life context, and the beginning-of-life context see respectively, for example: Stencel and Proszewska (2018, 606–607)—the philosophy of biology context; Nicholson (2014)—the philosophy of biology context; Shewmon (2001)—the end-of-life context; Brown (2019)—the beginning of life context.

5. Note that Shewmon himself believes that there might be at least several equally sound meanings of the word “death.” See: Shewmon and Shewmon (2004); Shewmon (2010). See also the discussion of Shewmon’s view on the meaning of the word “death”: Lizza (2018a).

6. For utilization of the notion of autopoiesis in the context of brain death debate see, for example, Shewmon (1985, 461) and Nguyen (2018a, 409–19).

7. Much more recently, work on computer programs fulfilling the criterion of autopoiesis has been advanced, for example, by Paul Bourgine and John Stewart (2004). However, some scholars disagree that computer programs might fulfill the criteria for autopoiesis (see, e.g., Luisi, 2003, 51; Nguyen, 2018a, 416–419). Such disagreement shows, in my view, that the notion of autopoiesis is not clear enough to delineate what counts as an autopoietic system and what does not.

8. Note, however, that homeostasis is not quantifiable on a continuous numerical scale, so it cannot be portrayed precisely as inversely proportional to entropy.

9. Such a view on death differs from the one developed originally by Erwin Schrödinger, who claimed that death is a state of the maximum entropy of an organism (1992, 69; cf. Nguyen 2018a, 379–387). The difference between the entropic concept of death as presented in this article and the original view amounts to the fact that, according to the view developed in the context of the brain death debate, death occurs earlier than according to Schrödinger’s idea. That is because the loss of the capacity to resist entropy occurs before the organism reaches the state of maximum entropy. The original entropic view on death can hardly be accepted, since even in the case of patients in a state of rigor mortis, not to mention patients with cessation of circulation, the statement that their organisms have reached a state of maximal entropy is, strictly speaking, false. That is because the state of maximum entropy is defined as a state “... in which no observable events occur” (Schrödinger, 1992, 69), and clearly the body in a state of rigor mortis still changes, as its putrefaction processes are progressing. Death, in this sense, is no more an event that separates the process of dying from the process of decay (as Bernat, Culver, and Gert stated that it should be; see 1981, 389), but rather is an ending point of the latter.

10. It is helpful to recall Lawrence Sklar’s distinction between nomologically irreversible processes and de facto irreversible processes here (1974, 359–394). The reversion of a nomologically irreversible process cannot happen since it is “forbidden” by the laws of physics, whereas the reversion of de facto irreversible process can happen, but it is extremely improbable. The only processes that are nomologically irreversible are weak interactions, and all others, including biological ones, are only de facto irreversible. This distinction is essential in the context of the entropic concept of death since it shows that there is no substantial difference in terms of the irreversibility of entropy increase between the case of hemophiliac as depicted above, and the other variants of such case in which hemophiliac would suffer some other injury. Another thing that is worth mentioning is the fact that the irreversibility of a condition might not be based solely on the internal properties of the objects: in the hemophiliac’s case, the irreversibility of the condition is partially settled by the set of external factors that amount to the fact that no one is available to provide help, see: Lizza (2018b, 79–81). Note as well that there is a substantial difference in terms of irreversibility between the hemophiliac case and that of the baseball player (described by Nair-Collins and Miller), whose condition could be reversed if we decide to provide CPR. See Nair-Collins and Miller (2016). Here in the hemophiliac case, there is no one around to help, while in the case of the baseball player, there was *no one who intended* to help.

11. Rare exceptions include recent works of Bertnat (2019), Huang and Bernat (2019).

12. There is at least one concept of an organism developed in the context of the brain death debate which is omitted in this article, namely the one developed by the President’s Council on Bioethics (2008) in their position two. In my opinion, this version of the biological concept of death has already been successfully criticized, and I do not see it necessary to summarize this criticism, nor to strengthen it here: see, for example, Shewmon (2009), Miller and Truog (2012, 72–75), Lizza (2018a; fn. 48), Nair-Collins (2018a, 35–36), Singer (2018, 158–161).

13. A somewhat similar observation is made by Shewmon: “Conception and deanimation mark the beginning and end of an organism as it is ‘in itself,’ whereas birth and passing away mark the beginning and end of an organism in relation to the rest of the world. The former could also be thought of as an organism’s ‘metaphysical’ beginning and end and the latter as its ‘civil’ beginning and end.” (2010, 276). Note, however, that Shewmon’s distinction between deamination and passing away hinges on the distinction between the irreversible and permanent cessation of an organism, and his view differs in this respect from that of Lizza (2018a, 9) or my own.

14. For a substantial discussion of Nair-Collins’ objections against the moral concept of death, see Nowak (2018).

15. The same objection is often raised against the concept of death, which identifies it with the loss of personal identity, such as elaborated by Michael Green and Daniel Wikler (1980).

16. My more developed moral concept of death can be found in Nowak (2018).

17. One might wonder why Warren adopts the last three of the mentioned criteria, since for her moral status is a matter of direct obligations. If we consider the criterion of being an object which might be cared for as an example, and abstract from any other capacities of this object, it seems that any infringement on such an entity is wrong just because it wrongs one who cares about this object, not because it wrongs the object itself. While I would personally sympathize with the idea of excluding the last three of the mentioned criteria from Warren’s list, it would, however, go too far from the main subject of this article to discuss this issue at full length. Therefore, in the following part of the article, I take the whole list of Warren’s criteria at face value. Another vital point to make here is to note that Warren’s six criteria that are incorporated here are not to be conflated with the criteria for personality, elaborated by her earlier work (1973, 55).

18. Note that although this paper criticizes the biological concept of death *as enshrined in the mainstream brain death debate*, it does not undermine all biological considerations about death. See the section: “Death as a moral rather than a purely biological issue.” Therefore, even if contrary to what I claim in this article, other entities than humans would completely lack any moral status, we can still talk about the death of animals and plants within a biological paradigm on the basis of the multiple concepts of an organism.

19. I use this term in the sense that was developed by McMahan (2002, 3–94). Note that the notion of “one of us” as McMahan uses it should not be equated with the much narrower and stringent notion of “person,” therefore the death of a human being cannot be identified with an irreversible loss of personal identity or with the irreversible loss of a status of a person. The reason that McMahan rejects the popular view that we are persons springs from the counterintuitive consequences of Derek Parfitt’s theory of personal identity. According to Parfitt’s theory, patients in end-stage dementia are not the same persons as before they reached this most profound level of memory injury, since they lack an appropriate level of psychological continuity as well as an appropriate level of psychological connections. Moreover, Parfitt’s theory implies that patients in end-stage-dementia are not persons whatsoever (not merely that they are not the same persons). Therefore, Parfitt’s approach entails that no person has egoistic reasons to care about her future as an end-stage-dementia-patient, since it will not be the future of her—the argument goes. This seems counterintuitive to McMahan and thus he proposes a different model of “who we are,” assuming that we are some sort of weaker psychophysical entities than persons—embodied minds. According to MacMahan, what is needed for our continued existence as the same embodied mind is “the continued existence and functioning, in nonbranching form, of enough of the same brain to be capable of generating consciousness or mental activity. This criterion stresses the survival of one’s basic psychological capacities, in particular the capacity for consciousness. It does not require continuity of any of the particular contents of one’s mental life. This allows that one may survive the deprogramming of one’s brain and that one continues to exist throughout the progress of Alzheimer’s disease, until the disease destroys one’s capacity for consciousness.” (2002, 68).

20. It is interesting to consider how the concepts of our identity and that of moral status intersect with a theory of a soul. In contrast to the moral concept of death that is presented in this article, one could claim that we are souls or that the soul is the sole criterion of our moral status. There are, however, numerous problems associated with the concept of a soul, as well described by McMahan (2002, 7–23). Theologians engaged in the discussion try to reach a consensus regarding the status of brain-dead patients. Yet, they are quite far from accomplishing this goal. For example, on the one hand, Patrick Lee (2016) upholds that the human soul cannot be present if a patient lacks “a radical capacity for conscious sensation,” claiming that the brain-dead lack this capacity. On the other hand, Doyen Nguyen forcefully argues that the human soul, as conceived within the Aristotelian tradition, is a unity. She observes that even if we can conceptually distinguish its powers such as vegetative, sensitive-locomotive, and intellective, the mere presence of activity of one such power in a human is sufficient proof of the presence of the whole human soul, including its intellective power (Nguyen, 2018b, 53). Since, according to Nguyen, we can observe the activity of the vegetative power of a soul in brain-dead individuals as their bodies can grow, heal wounds, and so on, brain-dead still possess whole human souls. Nguyen’s theological analysis of death connects the traditional view with the biological concept since she identifies souls with the principle of somatic integration. She writes that “the form, the soul, the principle of somatic integration, and the principle of life, are different ways of designating the same immaterial ‘entity’” (Nguyen, 2018b, 52). Similarly, Melisa Moschella associates the soul with the principle of somatic integration, yet she comes to different conclusions to Nguyen. According to Moschella (2016), what is crucial if we want to know whether a soul is present or absent is to ask whether the somatic integration we might observe is caused internally or externally. Only self-integration counts for the presence of a soul, according to Moschella, and there is no self-integration in brain-dead bodies (Nguyen disagrees about this last statement; see 2018a, 311–30). The discussion of the concept of death as a departure of the soul is crucial for Catholics and other Christians. Arguments for the existence of the soul have even been constructed quite recently. For example, for Rick Machuga the existence of the soul is the best explanation of our ability to understand the meanings of words (see 2002, 124–125). Yet this argument remains controversial since it is based on questionable assumptions, for example, regarding the philosophy of meaning (cf. Speaks, 2019). A full-blown discussion of arguments for the existence of the soul and so the analysis of the concept of death as the departure of the soul is unfortunately beyond the scope of the present research. Yet, I would like briefly to consider one problematic case in this regard (cf. Shewmon, 2010, 272–273). Notice that one of the recent achievements of modern science was the growth of mini-brain organoids from stem cells. Such organoids present electrical activity similar to that of preterm babies (Trujillo et al., 2019). Imagine that scientists in the future will be able to grow standard-size human brains in this way. Now, suppose that it would become standard “life-saving” therapy for patients that are currently labeled as “brain-dead” to provide them with new brains grown from their own stem cells. Would such a treatment really be life-saving for a given patient? Or perhaps it would create an entirely new human being, since a brain grown from stem cells would not resemble the former brain of the patient in terms of the structure of its neural connections (even though it would be genetically identical to the former one). Such a case is problematic for theological concepts of death. It is unclear, at least to me, how theological views would account for it. Such a case could be, however, easily counted as a case of creating new life through the moral view presented herein.

21. For a full-blown theoretical model of posthumous harm, and how we might understand its impact on the well-being of a once-living human, see Nowak (2018).

22. As evidenced in several pieces of research, the cerebral hemispheres may not be necessary for sentience (Shewmon, Holmes, and Byrne, 1999; Merker, 2007). Note, however, that this concerns only children, especially newborns, and the neuroplasticity of their brain structures mean they lack cerebral hemispheres. Moreover, since we are interested in the death of a particular patient, the mere fact that their brainstem could produce sentience (which is hardly plausible in the case of adults) is not proof of their life. It might be proof of life of some other embodied mind of a lower moral status than an adult human being. It is worth citing at length how McMahan refers to these kinds of cases: “Assume that in these cases the cerebral hemispheres were initially sufficiently formed to be capable of supporting consciousness. They were then destroyed, but structures capable of supporting consciousness were subsequently developed in the brainstem. If this is what happened, I believe that one mind, supported by the cerebral hemispheres, began to exist but was soon destroyed. Thereafter a new and different mind began to exist, supported by apparently unique structures in the brainstem. In short, these are cases in which two different and distinct minds were generated serially by one and the same brain, and in which two different conscious subjects serially inhabited the same body. To the best of our knowledge, nothing comparable to this has ever occurred in the case of an adult […]. But if this could happen in the case of an adult brain, the Embodied Mind Account of Identity would have the same implication: the original person whose hemispheres had been destroyed would have ceased to exist and would have been succeeded or supplanted in the same body by a different conscious subject (whose level of cognitive function would presumably be too low to warrant the label ‘person’)” (2002, 441).

## References

[CIT0001] Ad Hoc Committee of the Harvard Medical School to Examine the Definition of Brain Death. 1968. A definition of irreversible coma: Report of Ad Hoc Committee of the Harvard Medical School to Examine the Definition of Brain Death. Journal of the American Medical Association 205(6):337–40. 5694976

[CIT0002] Badeau, M. A. 2003. Artificial life: Organization, adaptation and complexity from the bottom up. Trends in Cognitive Sciences 7(11):505–12. 14585448 10.1016/j.tics.2003.09.012

[CIT0003] Baker, L. R. 2005. *When Do Persons Begin and End?* Distinguished Faculty Lecture at University of Massachusetts, Amherst, MA [On-line]. Available: http://people.umass.edu/lrb/files/bak05whebM.pdf (accessed January 12, 2020).

[CIT0004] Bernat, J. L. 2002. The biophilosophical basis of whole-brain death. Social Philosophy and Policy 19(2):324–42. 12678092 10.1017/s0265052502192132

[CIT0005] ———. 2006. The whole-brain concept of death remains optimum public policy. The Journal of Law, Medicine and Ethics 34(1):35–43. 10.1111/j.1748-720X.2006.00006.x 16489982

[CIT0006] ———. 2013. Life or death for the dead-donor rule? The New England Journal of Medicine 369(14):1289–91. 24088089 10.1056/NEJMp1308078

[CIT0007] ———. 2019. Refinements in the organism as a whole rationale for brain death. The Linacre Quarterly 86(4):347–58. 32431427 10.1177/0024363919869795 PMC6880069

[CIT0008] Bernat, J. L., C. M. Culver, and B. Gert. 1981. On the definition and criterion of death. Annals of Internal Medicine 94(3):389–94. 7224389 10.7326/0003-4819-94-3-389

[CIT0009] Bourgine, P., and J. Stewart. 2004. Autopoiesis and cognition. Artificial Life 10(3):327–45. 15245631 10.1162/1064546041255557

[CIT0010] Brown, H. R., W. Myrvold, and J. Uffink. 2009. Boltzmann’s H-theorem, its discontents, and the birth of statistical mechanics. Studies in History and Philosophy of Modern Physics 40(2):174–91.

[CIT0011] Brown, M. T. 2019. The somatic integration definition of the beginning of life. Bioethics 33(9):1035–41. 31452225 10.1111/bioe.12638

[CIT0012] Chiong, W. 2005. Brain death without definitions. The Hastings Center Report 35(6):20–30. 16396202

[CIT0013] Clark, E. 2010. The problem of biological individuality. Biological Theory 5(December):312–25.

[CIT0014] Condic, M. 2016. Determination of death: A scientific perspective on biological integration. Journal of Medicine and Philosophy 41(3):257–78. 27075193 10.1093/jmp/jhw004 PMC4889815

[CIT0015] Elliott, K. C. 2011. Direct and indirect roles for values in science. Philosophy of Science 78(2):303–24.

[CIT0016] Fellermann, H. 2011. Wet artificial life: The construction of artificial living systems. The frontiers collection. In Principles of Evolution, eds. H. Meyer-Ortmanns and S. Thurner, 261–80. Berlin, Germany: Springer.

[CIT0017] Global Observatory on Donation and Transplantation. 2018. *Data base* [On-line]. Available: http://www.transplant-observatory.org/export-database/ (accessed July 11, 2020)

[CIT0018] Green, M. B., and D. Wikler. 1980. Brain death and personal identity. Philosophy & Public Affairs 9(2):105–33. 11661844

[CIT0019] Grisez G. , and Boyle, J. M. 1979. Life and Death with Liberty and Justice. A contribution to the Euthanasia Debate. Notre Dame, London: University of Notre Dame Press.

[CIT0020] Huang, A. P., and J. L. Bernat. 2019. The organism as a whole in an analysis of death. Journal of Medicine and Philosophy 44(6):712–31. 31586180 10.1093/jmp/jhz025

[CIT0021] Jaworska, A., and J. Tannenbaum. 2014. Person-rearing relationships as a key to higher moral status. Ethics 124(2):242–71.

[CIT0022] ———. 2018. The grounds of moral status. In The Stanford Encyclopedia of Philosophy (Spring 2018 Edition), ed. N. Zalta [On-line]. Available: https://plato.stanford.edu/archives/spr2018/entries/grounds-moral-status/ (accessed January, 3 2020).

[CIT0023] Jensen, C. E., P. Sørensen, and K. D. Petersen. 2014. In Denmark, kidney transplantation is more cost-effective than dialysis. Danish Medical Journal 61(3):1–5. 24814915

[CIT0024] Kingma, E. 2019. Were you a part of your mother? Mind 128(511):609623–646.

[CIT0025] Korein, J. 1978. The problem of brain death: Development and history. Annals of the New York Academy of Sciences 315(1):1926–38. 10.1111/j.1749-6632.1978.tb50327.x 371483

[CIT0026] Lamb, D. 1985. Death, Brain Death and Ethics. London, United Kingdom: Croom Helm.

[CIT0027] Lambert, F. L. 2002. Disorder—A cracked crutch for supporting entropy discussions. Journal of Chemical Education 79(2):187–92.

[CIT0028] Lee, P. 2016. Total brain death and the integration of the body required of a human being. Journal of Medicine and Philosophy 41(3):300–14. 27097647 10.1093/jmp/jhw005 PMC4889816

[CIT0029] Lizza, J. P. 2011. Where’s Waldo: The “decapitation gambit” and definition of death. Journal of Medical Ethics 37(12):743–6. 21989065 10.1136/medethics-2011-100109

[CIT0030] ———. 2018a. Defining death: Beyond biology. Diametros 55:1–19.

[CIT0031] ———. 2018b. In defense of brain death: Replies to Don Marquis, Michael Nair-Collins, Doyen Nguyen and Laura Specker Sullivan. Diametros 55:68–9.

[CIT0032] Luhmann, N. 1995. Social Systems. Translated by J. Bednarz and D. Baecker. Stanford, CA: Stanford University Press.

[CIT0033] Luisi, P. L. 2003. Autopoiesis: A review and a reappraisal. Naturwissenschaften 90:49–59. 12590297 10.1007/s00114-002-0389-9

[CIT0034] Machuga, R. 2002. In Defense of Soul. Grand Rapids, MI: Brazos Press.

[CIT0035] Magnus, D. A. 2018. A defense of the dead donor rule. The Hastings Center Report 48(6):S36–42. 30584849 10.1002/hast.951

[CIT0036] Marquis, D. 2010. Are DCD donors dead? The Hastings Center Report 40(3):24–31. 10.1353/hcr.0.0270 20549866

[CIT0037] ———. 2018. Death is a biological phenomenon. Diametros 55:20–6.

[CIT0038] McMahan, J. 2002. The Ethics of Killing: Problems at the Margins of Life. New York, NY: Oxford University Press.

[CIT0039] McMullin, B., and F. J Varela. 1997. Rediscovering computational autopoiesis. In Fourth European Conference on Artificial Life, eds. P. Husbands and I. Harvey, 146–65. Cambridge, MA: MIT Press.

[CIT0040] Merker, B. 2007. Consciousness without a cerebral cortex: A challenge for neuroscience and medicine. Behavioral and Brain Sciences 30(1):63–81. 17475053 10.1017/S0140525X07000891

[CIT0041] Miller, F. G., and R. D. Truog. 2012. Death, Dying, and Organ Transplantation. Reconstructing Medical Ethics at the End of Life. Oxford, United Kingdom: Oxford University Press.

[CIT0042] Mingers, J. 1995. Self-Producing Systems: Implications and Applications of Autopoiesis. New York, NY: Springer.

[CIT0043] ———. 1997. A critical evaluation of Maturana’s constructivist family therapy. Systems Practice 10(2):137–51.

[CIT0044] Moschella, M. 2016. Deconstructing the brain disconnection-brain death analogy and clarifying the rationale for the neurological criterion of death. Journal of Medicine and Philosophy 41(3):279–99. 27095749 10.1093/jmp/jhw006 PMC4889817

[CIT0045] Nagel, T. 1979. Death. In: Mortal Questions, ed. T. Nagel, 1–10. Cambridge, United Kingdom: Cambridge University Press.

[CIT0046] Nair-Collins, M. 2017. Can the brain-dead be harmed or wronged? On the moral status of brain death and its implications for organ transplantation. Kennedy Institute of Ethics Journal 27(4):525–59. 29307880 10.1353/ken.2017.0041

[CIT0047] ———. 2018a. A biological theory of death: Characterization, justification, and implications. Diametros 55:27–43.

[CIT0048] ———. 2018b. The public’s right to accurate and transparent information about brain death and organ transplantation. The Hastings Center Report 48(6):S43–5. 10.1002/hast.953 30584864

[CIT0049] Nair-Collins, M., S. R. Green, and A. R. Sutin. 2015. Abandoning the dead donor rule? A national survey of public views on death and organ donation. Journal of Medical Ethics 41(4):297–302. 25260779 10.1136/medethics-2014-102229 PMC4392220

[CIT0050] Nair-Collins, M., and F. G. Miller. 2016. Is heart transplantation after circulatory death compatible with the dead donor rule? Journal of Medical Ethics 42(5):319–20. 26984898 10.1136/medethics-2016-103464

[CIT0051] Nguyen, D. 2018a. The New Definitions of Death for Organ Donation. A Multidisciplinary Analysis from the Perspective of Christian Ethics. Bern, Switzerland: Peter Lang.

[CIT0052] ———. 2018b. A holistic understanding of death: Ontological and medical considerations. Diametros 55:44–62.

[CIT0053] Nicholson, D. J. 2014. The return of the organism as a fundamental explanatory concept in biology. Philosophy Compass 9(5):347–59.

[CIT0054] Nowak, P. G. 2018. Brain death as irreversible loss of a human’s moral status. Ethics & Bioethics (in Central Europe) 8(3–4):167–78.

[CIT0055] Nowak, P. G., and A. Stencel. 2022. How many ways can you die? Multiple biological deaths as a consequence of the multiple concepts of an organism. Theoretical Medicine and Bioethics 43(2):127–54. 35859085 10.1007/s11017-022-09583-2 PMC9477939

[CIT0056] Pepper, J. W., and M. D. Herron. 2008. Does biology need an organism concept? Biological Reviews 83(4):621–7. 18947335 10.1111/j.1469-185X.2008.00057.x

[CIT0057] President’s Commission for the Study of Ethical Problems in Medicine and Biomedical and Behavioral Research. 1981. Defining Death: A Report on the Medical, Legal and Ethical Issues in the Determination of Death. Washington, D.C.

[CIT0058] President’s Council on Bioethics. 2008. Controversies in the Determination of Death: A White Paper of the President’s Council on Bioethics. Washington, D.C.: President’s Council on Bioethics.

[CIT0059] Robertson, J. A. 1999. The dead donor rule. The Hastings Center Report 29(6):6–14. 10641238

[CIT0060] Ruse, M. 1989. Do organisms exist? American Zoologist 29(3):1061–6.

[CIT0061] Schechtman, M. 1996. The Constitution of Selves. Ithaca, NY: Cornell University Press.

[CIT0062] Schrödinger, E. 1992. What is Life. The Physical Aspect of the Living Cell with Mind and Matter & Autobiographical Sketches. Cambridge, United Kingdom: Cambridge University Press.

[CIT0063] Shewmon, D. A. 1985. The metaphysics of brain death, persistent vegetative state, and dementia. The Thomist: A Speculative Quarterly Review 49(1):24–80. 10.1353/tho.1985.0040 11652632

[CIT0064] ———. 1998. Chronic “brain death”: Meta-analysis and conceptual consequences. Neurology 51(6):1538–45. 9855499 10.1212/wnl.51.6.1538

[CIT0065] ———. 2001. The brain and somatic integration: Insights into the standard biological rationale for equating “brain death” with death. The Journal of Medicine and Philosophy: A Forum for Bioethics and Philosophy of Medicine 26(5):457–78. 10.1076/jmep.26.5.457.3000 11588655

[CIT0066] ———. 2009. Brain death: Can it be resuscitated? The Hastings Center Report 39(2):18–24. 10.1353/hcr.0.0122 19388381

[CIT0067] ———. 2010. Constructing the death elephant: A synthetic paradigm shift for the definition, criteria, and tests for death. The Journal of Medicine and Philosophy: A Forum for Bioethics and Philosophy of Medicine 35(3):256–98. 10.1093/jmp/jhq022 20439358

[CIT0068] ———. 2018a. The case of Jahi McMath: A neurologist View. The Hastings Center Report 48(6):S74–6. 30584850 10.1002/hast.962

[CIT0069] ———. 2018b. Truly reconciling the case of Jahi McMath. Neurocritical Care 29(2):165–70. 30112685 10.1007/s12028-018-0593-x

[CIT0070] Shewmon, D. A., G. L. Holmes, and P. A. Byrne. 1999. Consciousness in congenitally decorticate children: Developmental vegetative state as self-fulfilling prophecy. Developmental Medicine and Child Neurology 41(6):364–74. 10400170 10.1017/s0012162299000821

[CIT0071] Shewmon, D. A., and E. S. Shewmon 2004. The semiotics of death and its medical implications. In Brain Death and Disorders of Consciousness. Advances in Experimental Medicine and Biology, eds. C. Machado, and D. A. Shewmon, 89–114, vol. 550. Boston, MA: Springer 10.1007/978-0-306-48526-8_8 15053428

[CIT0072] Singer, P. 1994. Rethinking Life and Death. The Collapse of our Traditional Ethics. New York, NY: St. Martin’s Press.

[CIT0073] ———. 2018. The challenge of brain death for the sanctity of life ethic. Ethics and Bioethics (in Central Europe) 8(3–4):153–65

[CIT0074] Sklar, L. 1974. Space, Time, and Spacetime. Berkeley, CA: University of California Press.

[CIT0075] Speaks, J. 2019. Theories of meaning. In The Stanford Encyclopedia of Philosophy,ed. E. N. Zalta [On-line]. Available: https://plato.stanford.edu/archives/win2019/entries/meaning/ (accessed May 1, 2020).

[CIT0076] Stencel, A., and A. M. Proszewska. 2018. How research on microbiomes is changing biology: A discussion on the concept of the organism. Foundation of Science 23(4):603–20.

[CIT0077] Sulllins, J. P. 2005. Ethics and artificial life: From modeling to moral agents. Ethics and Information Technology 7(139):139–48.

[CIT0078] Symons, X., and R. M. Chua. 2018. Organismal death, the dead donor rule and the ethics of vital organ procurement. Journal of Medical Ethics 44(12):868–71. 29921617 10.1136/medethics-2018-104796

[CIT0079] Teubner, G. 1993. Laws as an Autopoietic System. Oxford, United Kingdom: Blackwell.

[CIT0080] Trujillo, C. A., R. Gao, P. D. Negraes, J. Gu, J. Buchanan, S. Preissl, A. Wang, et al. 2019. Complex oscillatory waves emerging from cortical organoids model early human brain network development. Cell Stem Cell 25(4):558–569.e7. 31474560 10.1016/j.stem.2019.08.002 PMC6778040

[CIT0081] Truog, R. D. 2018. Lessons from the case of Jahi McMath. The Hastings Center Report 48(6):S70–3. 10.1002/hast.961 30584856

[CIT0082] Varela, F. J. 1979. Principles of Biological Autonomy. New York, NY: North Holland.

[CIT0083] Varela, F. J., H. R. Maturana, and R. Uribe. 1974. Autopoiesis: The organization of living systems, its characterization and a model. Biosystems 5(4):187–96. 10.1016/0303-2647(74)90031-8 4407425

[CIT0084] Veatch, R. M. 1975. The whole-brain-oriented concept of death: An outmoded philosophical formulation. Journal of Thanatology 3(1):13–30. 11662185

[CIT0085] ———. 2003. The dead donor Rule: True by definition. The American Journal of Bioethics 3(1):10–11. 12859828 10.1162/152651603321611791

[CIT0086] ———. 2015. Killing by organ procurement: Brain-based death and legal fictions. Journal of Medicine and Philosophy 40(3):289–311. 25889264 10.1093/jmp/jhv007

[CIT0087] Veatch, R. M., and L. F. Ross 2016. Defining Death. The Case for Choice. Washington, DC: Georgetown University Press.

[CIT0088] Warren, M. A. 1973. On the moral and legal status of abortion. The Monist 57(1):43–61. 11661013 10.5840/monist197357133

[CIT0089] ———. 1997. Moral Status. Obligations to Persons and Other Living Things. Oxford, United Kingdom: Oxford University Press.

[CIT0090] Żuradzki, T., and P. G. Nowak. 2019. Withdrawal aversion as a useful heuristic for critical care decisions. American Journal of Bioethics 19(3):36–8. 10.1080/15265161.2018.1563656 31543054

